# Supplementary material for: Implementing digital computing with DNA-based switching circuits
Source: Nat Commun. 2020 Jan 8;11:121. doi: 10.1038/s41467-019-13980-y (PMC6949259; doi:10.1038/s41467-019-13980-y)
Supplement: Supplementary file 1 — Supplementary information [file 41467_2019_13980_MOESM1_ESM.pdf]

Supplementary Materials for

Implementing digital computing with DNA-based switching circuits

Fei Wang,[1,2†] Hui Lv,[3,6†] Qian Li,[1] Jiang Li,[3,4] Xueli Zhang,[2] Jiye Shi,[1] Lihua Wang,[*3,4,5] Chunhai Fan[*1]

[1]School of Chemistry and Chemical Engineering, and Institute of Molecular Medicine, Renji Hospital, School of Medicine, Shanghai Jiao Tong University, Shanghai 201240, China
[2]Joint Research Center for Precision Medicine, Shanghai Jiao Tong University & Affiliated Sixth Peoples Hospital South Campus, Southern Medical University Affiliated Fengxian Hospital, Shanghai 201499, China
[3]Division of Physical Biology, CAS Key Laboratory of Interfacial Physics and Technology, Shanghai Synchrotron Radiation Facility, Shanghai Institute of Applied Physics, Chinese Academy of Sciences, Shanghai 201800, China
[4]Zhangjiang Laboratory, Shanghai Advanced Research Institute, Chinese Academy of Sciences, Shanghai 201210, China
[5]Shanghai Key Laboratory of Green Chemistry and Chemical Processes, School of Chemistry and Molecular Engineering, East China Normal University, 500 Dongchuan Road, Shanghai, 200241, China
[6]University of Chinese Academy of Sciences, Beijing 100049, China
*Correspondence to: wanglihua@sinap.ac.cn; fanchunhai@sjtu.edu.cn
†These authors contributed equally to this work

Table of Contents

Supplementary Methods Page 2

Supplementary Figures 1-15 Page 3-11

Supplementary Table 1-5 Page 12-16

Supplementary References Page 17

Supplementary Methods

Materials

The DNA oligonucleotides used in this study were purchased from Sangon Biotech (Shanghai). Unlabeled DNA oligonucleotides were purified by Sangon using ULPAGE, and labeled DNA oligonucleotides were purified by Sangon using HPLC. Individual unlabeled DNA oligonucleotides were dissolved in 1×TE buffer (nuclease free, pH 8.0, Sigma-Aldrich), quantified by A260 using UV/Vis spectrometry and stored at -20℃. Oligos labelled with dyes or quenchers were dissolved in deionized water (Milli-Q), quantified by A260 and stored in deionized water at -20℃.

Methods

Preparation of a computing circuit.

To prepare a downstream switch molecule, s1, s2 and s3 were mixed in Tris-EDTA buffer (1× Tris-EDTA: 40 mM Tris base, 20 mM acetic acid, 2 mM EDTA adjusted to pH 8.0) with 12.5 mM $MgCl_2$ to final concentrations of 11 μM ,10 μM and 15 μM respectively (Supplementary Fig. 1). To prepare a starting switch molecule, s1 and s2 were mixed in Tris-EDTA buffer to final concentration of 11 μM and 10 μM respectively. Quencher strand and fluorescence strand were mix at molar ratio of 1.5:1 in Tris-EDTA buffer with 12.5 mM $MgCl_2$ to 10 μM. The reaction mix was then annealed by heating to 95 ℃ for 2 min and slowly cooling to room temperature at 0.1 ℃ every 6 s, then held at 4 ℃. The hybridized molecules were stored at 4 ℃ for further use.

Numerical simulation of reaction kinetics

Models of switching circuit (Supplementary Fig. 2a) and logic gate circuit (Supplementary Fig. 2b) were built using SimBiology Tool in Matlab. Desired reaction rate and unwanted leakage rate were considered for each reaction. MassAction was used as KineticLaw for all reactions. To theoretically understand the difference of logic gate circuit and switching circuit architectures, we used universal reaction rate and leakage rate. And we used the same reactions rate for logic gate circuit and switch circuit (Supplementary Table 5). The output kinetics were simulated with all possible input combinations.

Supplementary Figures

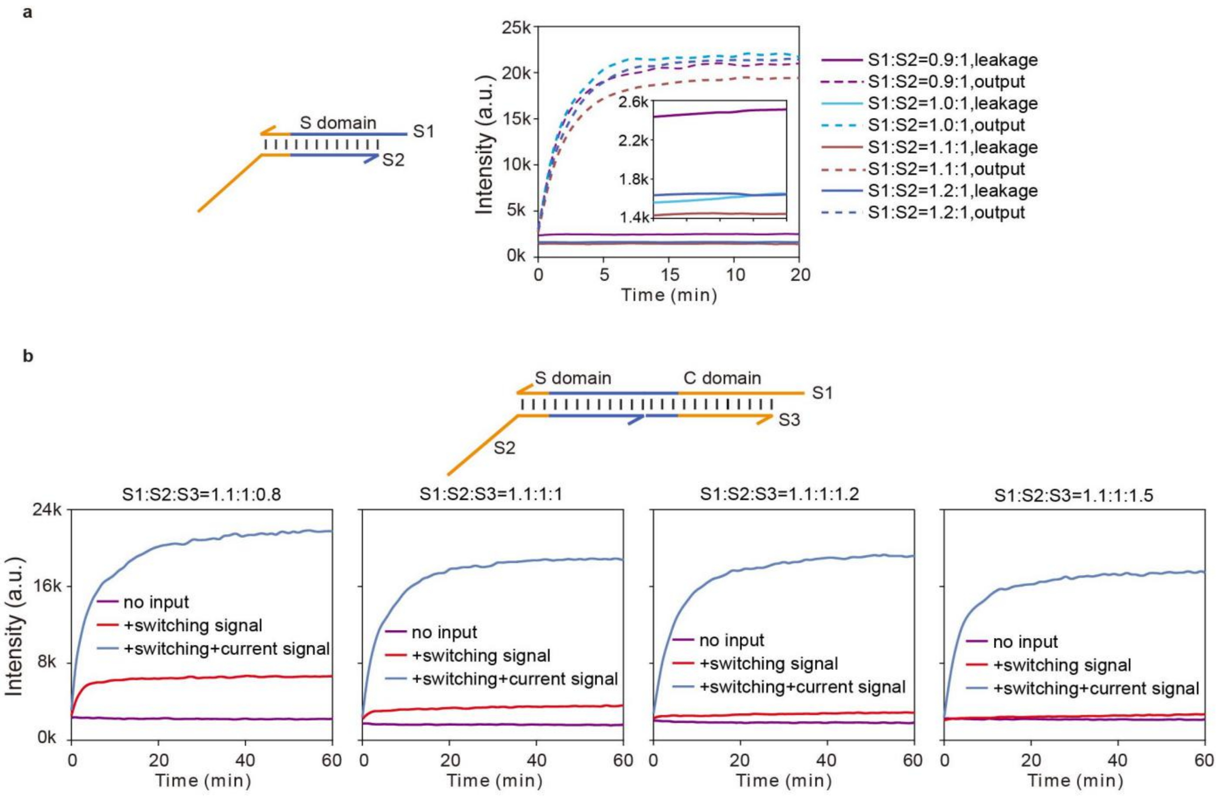

Supplementary Figure 1. Optimization of ratio between strands within a switch. a, Switching performance with different concentration ratios between S1 and S2 in a starting switch. A higher concentration of S1 ensures complete hybridization of S2, decreasing signal leakage. As 1.1× is sufficient for suppressing leakage, we used S1:S2=1.1:1 for experimental tests. **b,** Switching performance with different S3 concentrations in a downstream switch. Excessive S3 helps suppress leakage caused by unwanted switch flipping by switching signal at the absence of current signal (red line). So we used the ratio S1:S2:S3=1.1:1:1.5 for experimental tests. Source data are provided as a Source Data file.

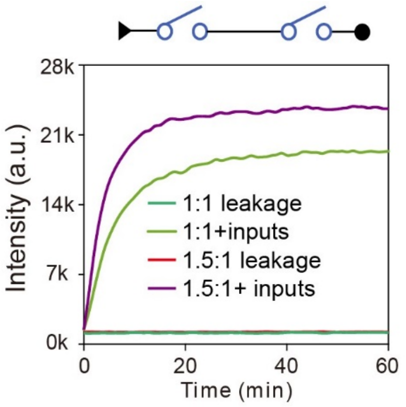

Supplementary Figure 2. Output and leakage with different ratios of upstream switch and downstream switch. To ensure that the upstream switch could produce sufficient output for the downstream switch, we used a ratio of 1.5:1. Source data are provided as a Source Data file.

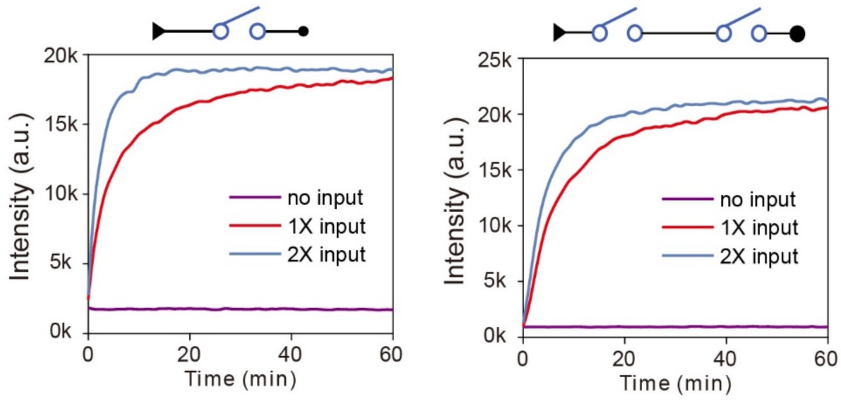

Supplementary Figure 3. Switching performance with 1× and 2× inputs. For single-switch and layered-switch circuits, 2× input could result in quicker switch flipping and higher plateau. So we used 2× switching signals as inputs. Source data are provided as a Source Data file.

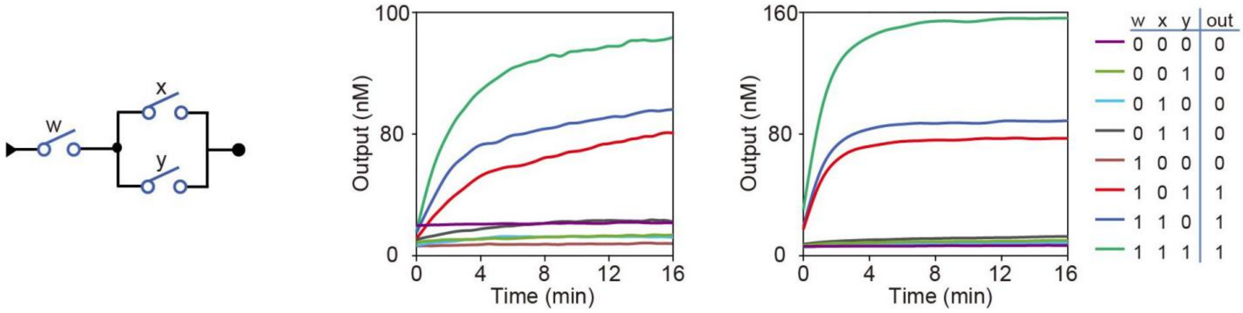

Supplementary Figure 4. Optimization of upstream switch and downstream switch molar ratio for signal splitting circuits. The signal splitting circuit produces only half of the output at a S(w): CS(x): CS(y) molar ratio of 1:1:1 (middle), so a ratio of 3:1:1 was used considering signal splitting and signal decay caused by effective concentrations (right). Source data are provided as a Source Data file.

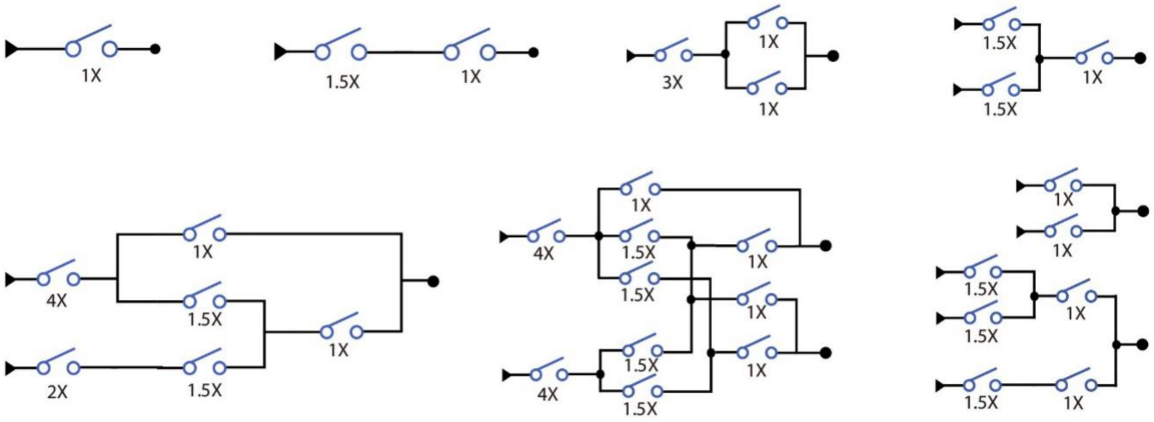

Supplementary Figure 5. Concentrations for the tested circuits. Here 1X means 100 nM.

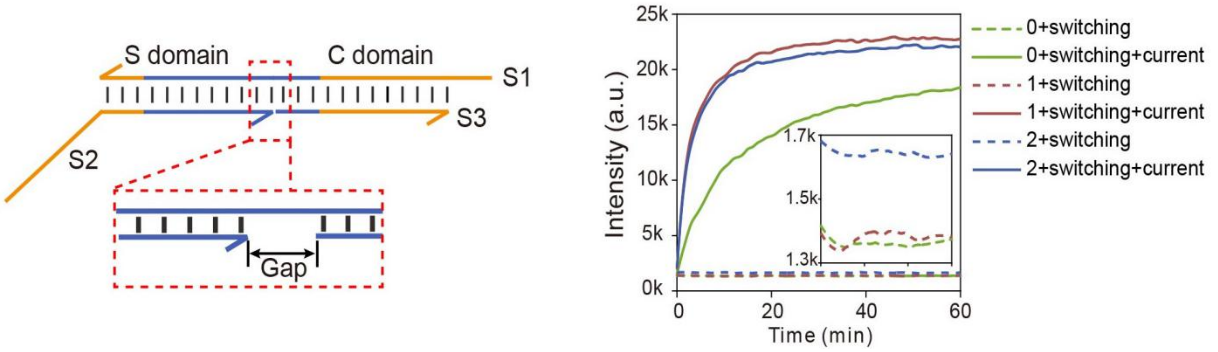

Supplementary Figure 6. Structural optimization of the downstream switch. A downstream switch contains a C domain for current signal and a S domain for switching signal, which is formed by hybridization of three strands. The required features are quick replacement of S3 by current signal and low leakage from replacement of S2 by switching signal at the absence of current signal. We found a 0 nt gap led to slow reaction and a 2 nt gap led to higher leakage. We chose 1 nt as the gap length which has low leakage similar to the 0 nt gap and high reaction rate identical to the 2nt gap. Source data are provided as a Source Data file.

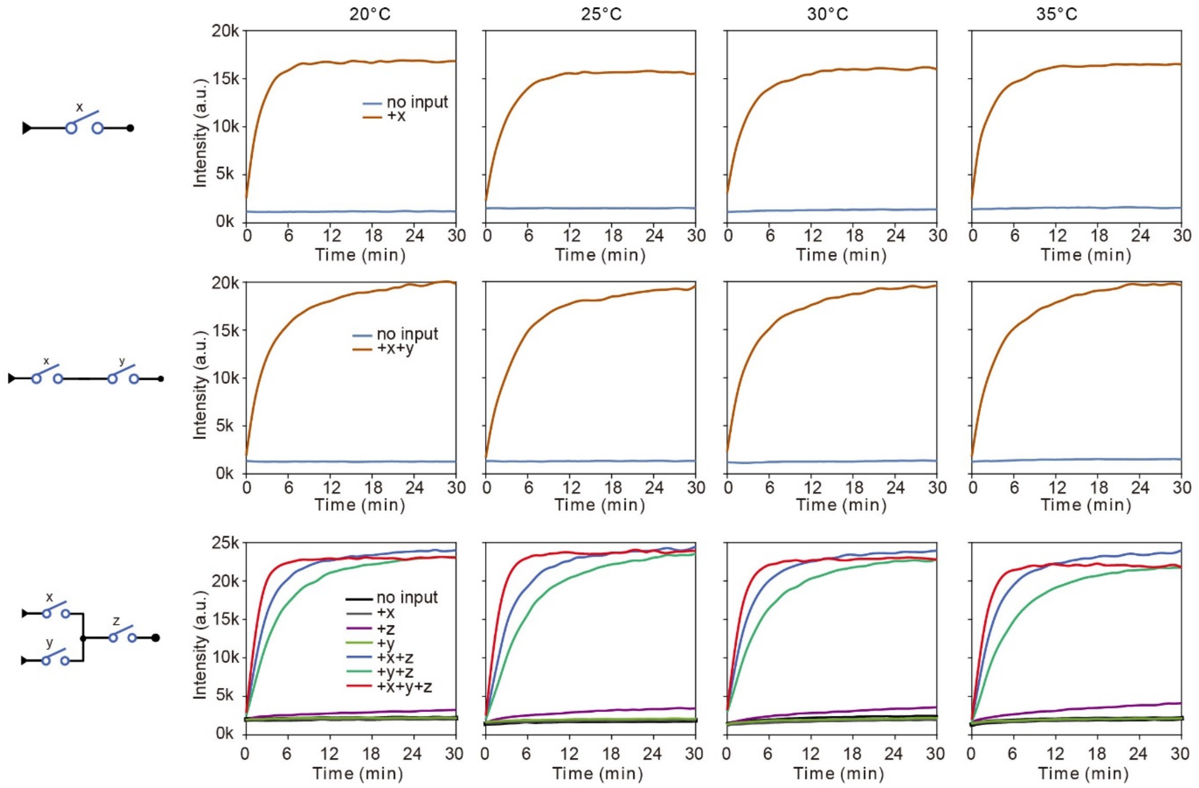

Supplementary Figure 7. The influence of reaction temperature on the operation of DSCs. Increasing temperature from 20 °C to 35 °C did not have significant influence on the leakage and output of the signal-switch and two-switch circuits. Despite the leakage for a fan-out circuit slightly increased with the temperature, it remained at low level. Source data are provided as a Source Data file.

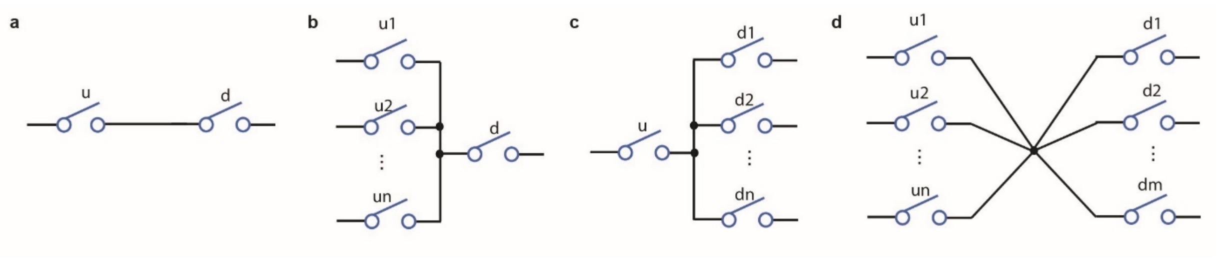

Supplementary Figure 8. Modular sequence design for possible adjacent connection patterns within a DSC. a, For the pattern that an upstream switch (u) followed by one downstream switch (d), the sequence design constraint is determined by C(d)=cs(u), where C(d) means C domain of d and cs(u) means current signal from u. b, For a fan-in pattern that n upstream switches are followed by one downstream switch, the constraints are determined by C(d)=cs(u1) =cs(u2)=…=cs(un). c, For a fan-out pattern that one upstream switch is followed by n downstream switches, the constraints are determined by C(d1)= C(d2)=…= C(dn)=cs(u). d, For a pattern that n upstream switches are followed by m downstream switches, the constraints are determined by C(d1)= C(d2)=…= C(dm)= cs(u1) =cs(u2)=…=cs(un).

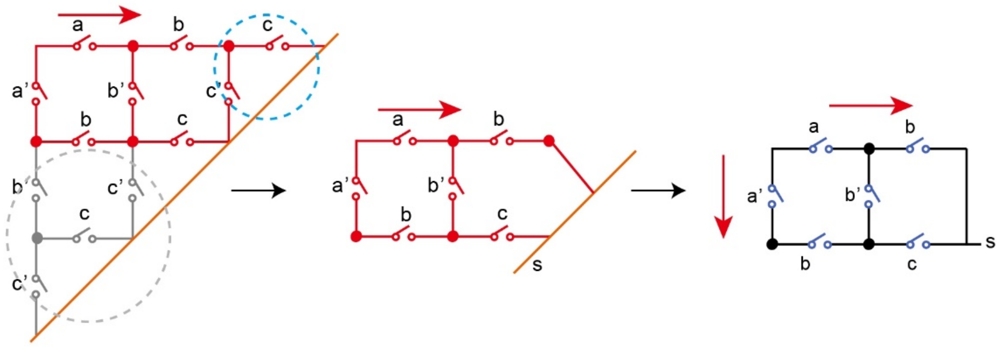

Supplementary Figure 9. Circuit simplification with on the switch canvas. The needed paths are show in red, and the grey part are not involved in the computing, which could be deleted. Then a complementary switch pair that links directly to reporter was replaced with a wire.

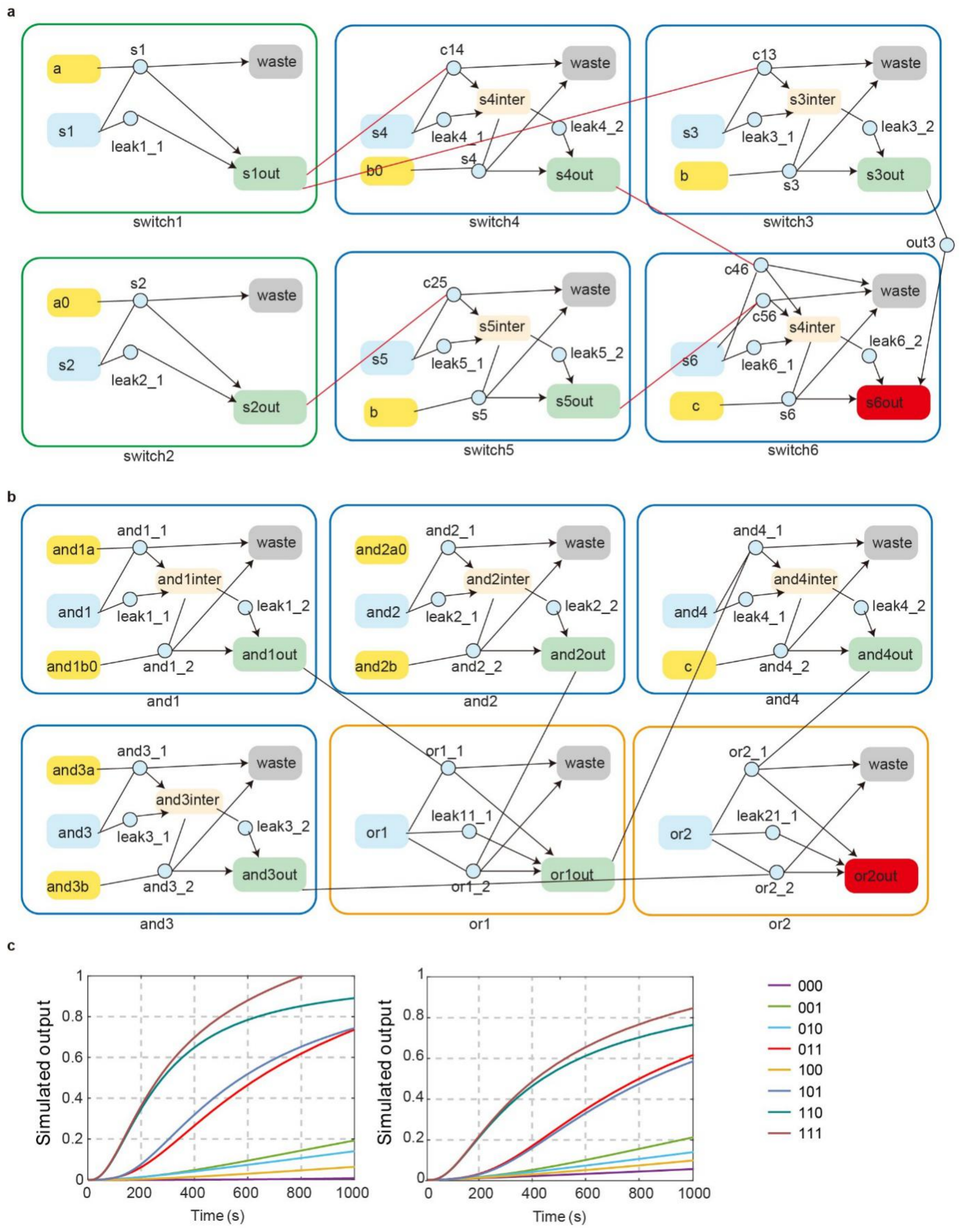

Supplementary Figure 10. Numerical simulation of the three-input voting function implemented with switching circuit and logic gate circuit. a, Diagram illustration of switching logic gate circuit. **b**, Diagram illustration of logic gate circuit. **c**, Simulated output for switching circuit (left) and logic gate circuit (right) for all possible input combinations. Source data are provided as a Source Data file.

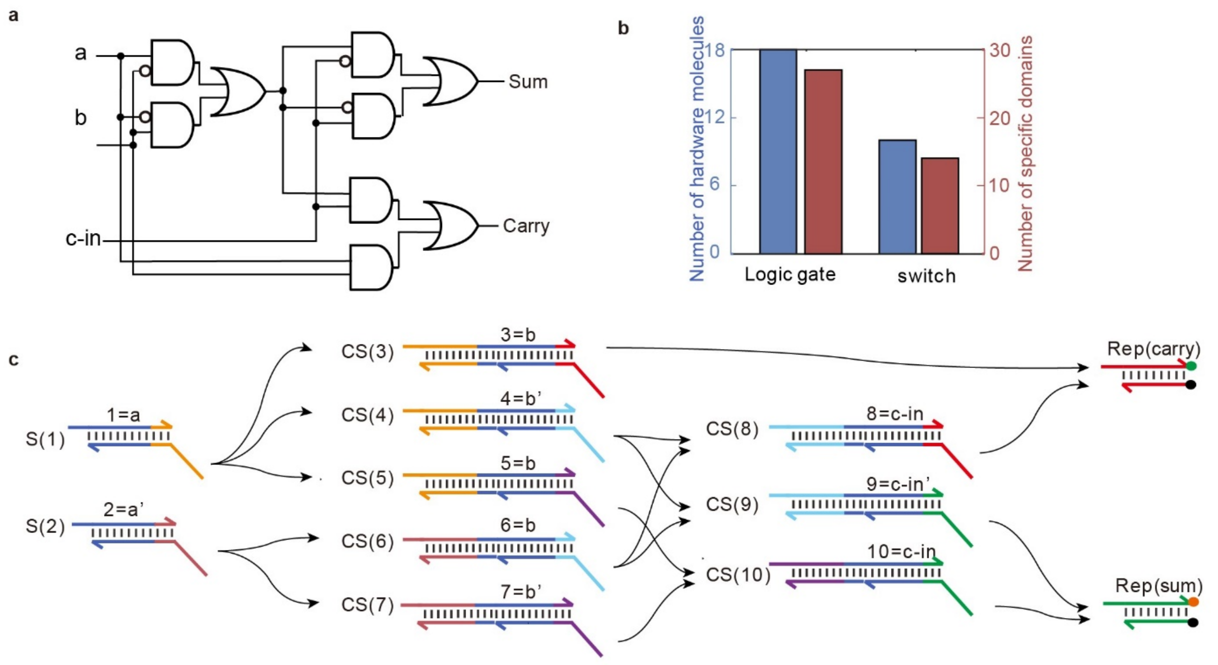

Supplementary Figure 11. Logic gate diagram and DSC implementation of full-adder. a, AND-OR logic gate circuit for full-adder function. **b**, Number of hardware molecules (left axis) and specific domains (right axis) used to realize the full-adder function with logic gates and switches respectively. **c**, Molecular implementation of the full-adder circuit with DCS. Source data are provided as a Source Data file.

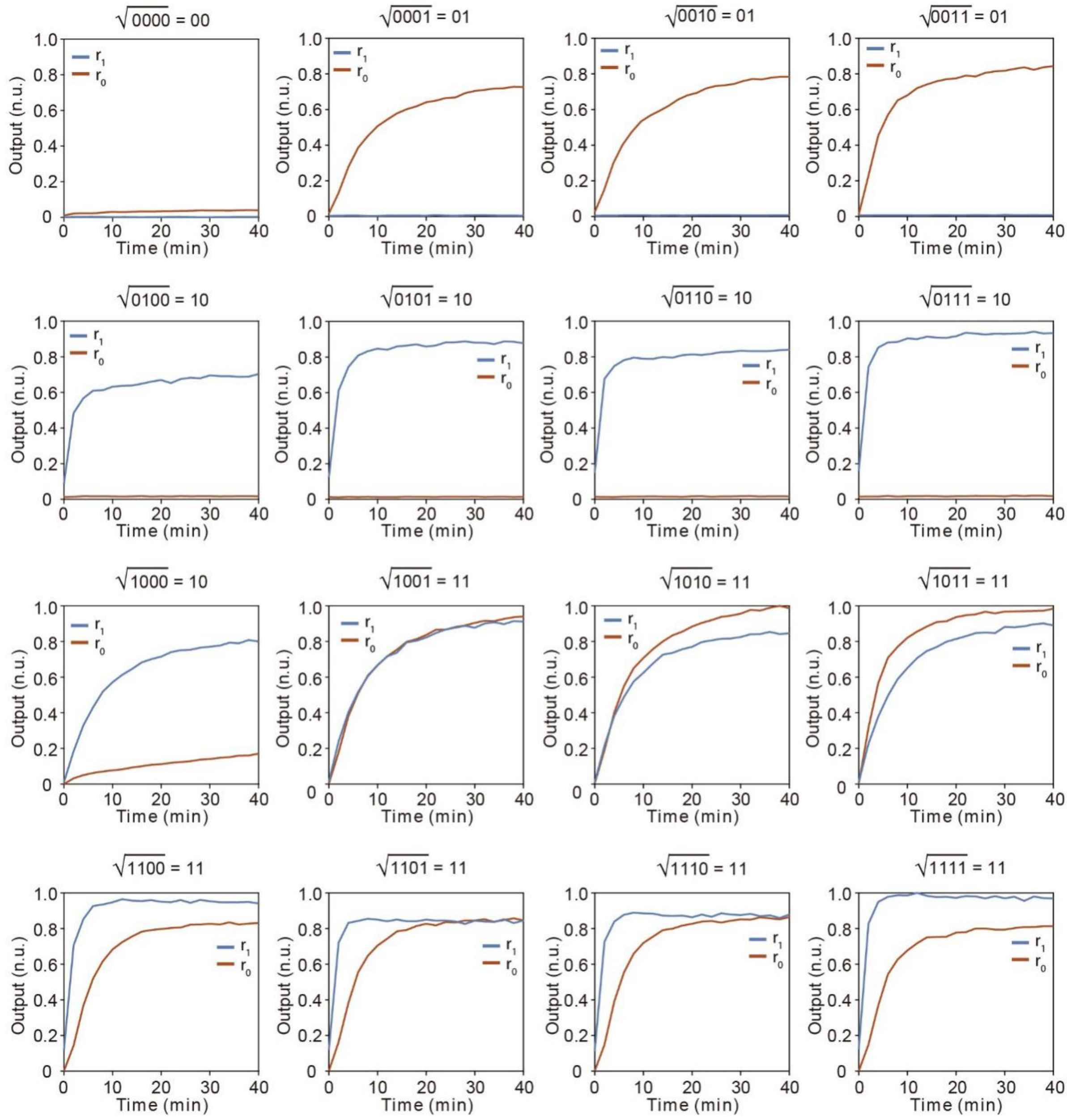

Supplementary Figure 12. Computing kinetics for all four-bit inputs with the square-root circuit. Source data are provided as a Source Data file.

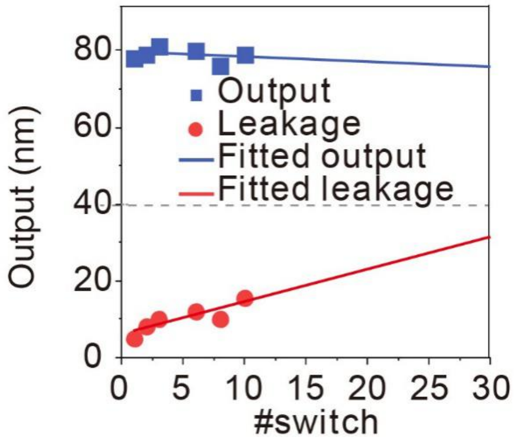

Supplementary Figure 13. Performance evaluation of switching circuits. The trend of output decay (blue line) and leakage increase (red line) with the increase of participated switches. A threshold of 40% suggests more than 30 switches could be used in a single reaction using our design. Source data are provided as a Source Data file.

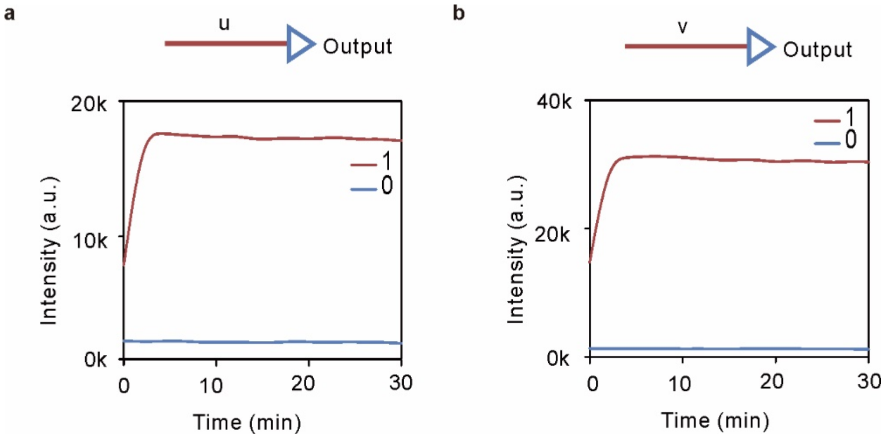

Supplementary Figure 14. Signal readout kinetics. a, Fluorescence data of interaction between reporter-opening strand and Reporter1. **b**, Fluorescence data of interaction between reporter-opening strand and Reporter2. Source data are provided as a Source Data file.

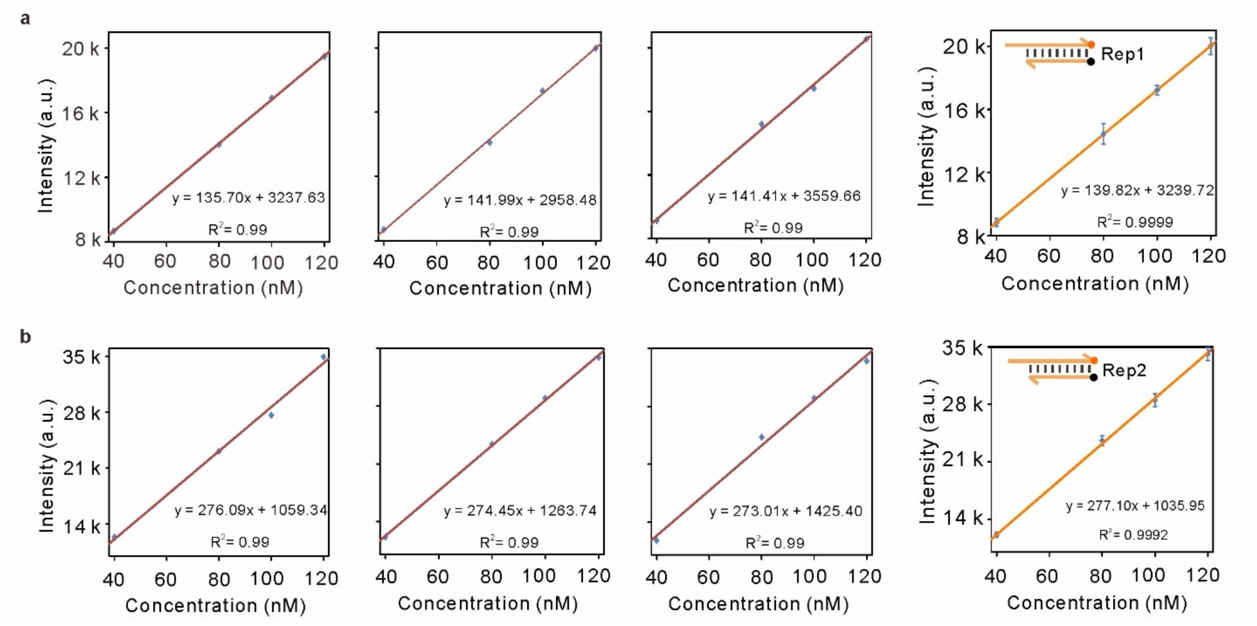

Supplementary Figure 15. Calibration curve for data normalization. a, Calibration curve for reporter 1. **b**, Calibration curve for reporter 2. The plots represent increase in fluorescent signal (blue square dots) due to unquenching of fluorophores in Reporter complexes upon addition of fixed concentrations of opening strands (40, 80, 100 and 120 nM) for each cuvette. The equations at the Inset represent the linear fits of the dots (with R-squared values) and are used to convert fluorescence units to concentration values for a specific Reporter in actual experiments. The right most maps are the average of the other three maps, respectively. Error bars represent s.d.. Source data are provided as a Source Data file.

Supplementary Tables

Supplementary Table 1. Computing speed of DSCs and logic gate implementations

**Reported work**	**Function**	**Computing speed ($t_{1/2}$)**
Qian et al. (2011) (*13*)	Square root (12 gates)	~ 8 hours
Seelig et al. (2006) (*1*)	Logic function (11 gates)	> 10 hours
Li et al. (2016) (*4*)	Half adder (2 gates)	~6 hours
Current work	Full adder (10 switches)	~ 10 minutes

Supplementary Table 2. Oligos used in each function

**Function**	**Oligos used**
Figure 2c S(x)	dc-01
Figure 2d S(x);CS(y)	dc-02; dc-03
Figure 3b S(w);CS(x);CS(y)	dc-02; dc-03; dc-05
Figure 3e S(x); S(y); CS(z)	dc-02; dc-06; dc-03
Figure 4 S(1); S(2); CS(3); CS(4); CS(5); CS(6);	dc-02; dc-19; dc-03; dc-07; dc-08; dc-10
Figure 6 a_3; a_2 (top), a_1; a_0; a_2'; a_2; a_3 (bottom)	dc-31; dc-32 (top), dc-02; dc-06; dc-03; dc-30; dc-10 (bottom)
Figure S11 S(1); S(2); CS(3); CS(4); CS(4); CS(5); CS(6);CS(7); CS(8); CS(9); CS(10)	dc-02; dc-19; dc-03; dc-07; dc-11; dc-08; dc-13; dc-10; dc-12; dc-14
Figure S14 u,v	dc-14-2; dc-03-2
Rep1	Rep (sum)
Rep2	Rep (carry)
Figure S1a; S1b	dc-01; dc-03
Figure S2	dc-02; dc-03
Figure S3 left, right	dc-01 (left), dc-02; dc-03 (right)
Figure S4 w; x; y	dc-02; dc-03; dc-05
Figure S6	dc-03; dc03-3-0gap; dc-03-3-2gap
Figure S7 top, middle, bottom	dc-01 (top), dc-02; dc-03 (middle), dc-02; dc-06; dc-03 (bottom)

Supplementary Table 3. DNA sequence of DSCs

No.	Sequences Name	Sequences (5`- 3`)
**1**	dc-01-1	GAGTAGATTGGTGAATAGTTGAGATG
**2**	dc-01-2	TCCACTATCTTCCTCATCTCAACTATTCACCAATC
**3**	dc-in01-1	CATCTCAACTATTCACCAATCTACTC
**4**	dc-02-1	AGTAGGATTGGTGAATAGTTTAGGGA
**5**	dc-02-2	CAAACTCCTAATACTCCCTAAACTATTCACCAATC
**6**	dc-in02-1	TCCCTAAACTATTCACCAATCCTACT
**7**	dc-03-1	TTAGGGAGTATTAGGAGTTTGGAGTAGAGATGTGGTAGAGT GAGATG
**8**	dc-03-2	TCCACTATCTTCCTCATCTCACTCTACCACATCTC
**9**	dc-03-3	ACTCCAAACTCCTAATACTC
**10**	dc-in03-1	CATCTCACTCTACCACATCTCTACTC
**11**	dc-04-1	GAGTAGAAGATGATGAAGATGAGATG
**12**	dc-04-2	TCCACTATCTTCCTCATCTCATCTTCATCATCTTC
**13**	dc-in04-1	CATCTCATCTTCATCATCTTCTACTC
**14**	dc-05-1	TTAGGGAGTATTAGGAGTTTGAGTGAGAATGTATGGTTTGTG AGATG
**15**	dc-05-2	TCCACTATCTTCCTCATCTCACAAACCATACATTC
**16**	dc-05-3	CACTCAAACTCCTAATACTC
**17**	dc-in05-1	CATCTCACAAACCATACATTCTCACT
**18**	dc-06-1	GAGTAGAATGAATGAAGTGTTAGGGA
**19**	dc-06-2	CAAACTCCTAATACTCCCTAACACTTCATTCATTC
**20**	dc-in06-1	TCCCTAACACTTCATTCATTCTACTC
**21**	dc-07-1	TTAGGGAGTATTAGGAGTTTGTGGAAGAGTTAGGTTGAAGG GTTAGA
**22**	dc-07-2	CACCAATTCAAACTTCTAACCCTTCAACCTAACTC
**23**	dc-07-3	TCCACAAACTCCTAATACTC
**24**	dc-in07-1	TCTAACCCTTCAACCTAACTCTTCCA
**25**	dc-08-1	AGGGTGAGTAATTTGTGTGTGGGTTAGAAATAATGGAAGGG GTTAGA
**26**	dc-08-2	CACCAATTCAAACTTCTAACCCCTTCCATTATTTC
**27**	dc-08-3	AACCCACACACAAATTACTC
**28**	dc-in08-1	TCTAACCCCTTCCATTATTTCTAACC
**29**	dc-10-1	GGTTAGAAGTTTGAATTGGTGAATAAGAGGAGAGTAATGGT GAGATG
**30**	dc-10-2	TCCACTATCTTCCTCATCTCACCATTACTCTCCTC

**31**	dc-10-3	TATTCACCAATTCAAACTTC
**32**	dc-in10-1	CATCTCACCATTACTCTCCTCTTATT
**33**	dc-11-1	TTAGGGAGTATTAGGAGTTTGTAAGAGATAGGTAGGTGGAG TTGGGA
**34**	dc-11-2	CAATCAAATTCCACTCCCAACTCCACCTACCTATC
**35**	dc-11-3	CTTACAAACTCCTAATACTC
**36**	dc-in11-1	TCCCAACTCCACCTACCTATCTCTTA
**37**	dc-12-1	GGTTAGAAGTTTGAATTGGTGTTAGAGAGGTTAATGGTGAG AGTAGA
**38**	dc-12-2	CACCACTCAATCCTTCTACTCTCACCATTAACCTC
**39**	dc-12-3	CTAACACCAATTCAAACTTC
**40**	dc-in12-1	TCTACTCTCACCATTAACCTCTCTAA
**41**	dc-13-1	AGGGTGAGTAATTTGTGTGTGTTTAAGAAAGTTGGATTGTGT TGGGA
**42**	dc-13-2	CAATCAAATTCCACTCCCAACACAATCCAACTTTC
**43**	dc-13-3	TAAACACACACAAATTACTC
**44**	dc-in13-1	TCCCAACACAATCCAACTTTCTTAAA
**45**	dc-14-1	GTTGGGAGTGGAATTTGATTGTGTGAGAGTATTTAGTTGGGA GTAGA
**46**	dc-14-2	CACCACTCAATCCTTCTACTCCCAACTAAATACTC
**47**	dc-14-3	CACACAATCAAATTCCACTC
**48**	dc-in14-1	TCTACTCCCAACTAAATACTCTCACA
**49**	dc-19-1	GGAATGAGAAAGTGTAAGAAGGGTGA
**50**	dc-19-2	CACACACAAATTACTCACCCTTCTTACACTTTCTC
**51**	dc-in19-1	TCACCCTTCTTACACTTTCTCATTCC
**52**	dc-30-1	CACGTGAGAAAGTGTAAGAGGAATGA
**53**	dc-30-2	CACCAATTCAAACTTCTAACCTCTTACACTTTCTC
**54**	dc-in30-1	TCTAACCTCTTACACTTTCTCACGTG
**55**	dc-31-1	TGCACTGTGTTGAAGGTGTGAGTAGA
**56**	dc-31-2	CACCACTCAATCCTTCTACTCACACCTTCAACACA
**57**	dc-in31-1	TCTACTCACACCTTCAACACAGTGCA
**58**	dc-32-1	GAACGTGTTGAAATGTGGTGAGTAGA
**59**	dc-32-1	CACCACTCAATCCTTCTACTCACCACATTTCAACA
**60**	dc-in32-1	TCTACTCACCACATTTCAACACGTTC
**61**	dc03-3-0gap	TACTCCAAACTCCTAATACTC
**62**	dc-03-3-2gap	CTCCAAACTCCTAATACTC
**63**	rep1-1	GAGTAGAAGGATTGAGTGGTG-3`TET
**64**	rep1-2	5`BHQ2-CACCACTCAATCCTTC

**65**	rep2-1	TGAGATGAGGAAGATAGTGGA-3`TET
**66**	rep2-2	5`BHQ1-TCCACTATCTTCCTCA

Supplementary Table 4. DNA sequence of logic gate circuit

No.	Sequences Name	Sequences (5`- 3`)
**1**	L-21-1	AGTGAGAATGTATGGTTTATGAGATG
**2**	L-21-2	TCCACTATCTTCCTCATCTCATAAACCATACATTC
**3**	L-15-1	GAGTAGAGTAATTTGTGTGTGTGTAAGAAAGTTGGAGTGAGAAT GTA
**4**	L-15-2	CATCTCATAAACCATACATTCTCACTCCAACTTTC
**5**	L-15-3	TACACACACACAAATTACTC
**6**	L-in15-1	TACATTCTCACTCCAACTTTCTTACA
**7**	L-in15-0	CAACTTCCTTAAACTCTACTC
**8**	L-20-1	GAGTAGAGAAAGTGTAAGAGAGTAGA
**9**	L-20-2	CACACACAAATTACTCTACTCTCTTACACTTTCTC
**10**	L-16-1	GAGTAGAGTATTAGGAGTTTGTAAGAGATAGGTAGGAGTAGAGA AAG
**11**	L-16-2	TCTACTCTCTTACACTTTCTCTACTCCTACCTATC
**12**	L-16-3	CTTACAAACTCCTAATACTC
**13**	L-in16-2-1	CAAACTCCTAATACTCTACTC
**14**	L-in16-1-1	CTTTCTCTACTCCTACCTATCTCTTA
**15**	L-in16-0	CATACACTAACTCTTCTACTC
**16**	L-17-1	AGTAGGAAGTTTGAATTGGTGTTAGAGAGGTTAATGAGTAGAGA AAG
**17**	L-17-2	TCTACTCTCTTACACTTTCTCTACTCATTAACCTC
**18**	L-17-3	CTAACACCAATTCAAACTTC
**19**	L-in17-2-1	CACCAATTCAAACTTCCTACT
**20**	L-in17-1-1	CTTTCTCTACTCATTAACCTCTCTAA
**21**	L-in17-0	CATCTCATCACCATTAACCTC
**22**	L-18-1	GGAATGAGTGGAATTTGATTGGAGTAGAGTATTTAAGTGAGAAT GTA
**22**	L-18-2	CATCTCATAAACCATACATTCTCACTTAAATACTC
**23**	L-18-3	ACTCCAATCAAATTCCACTC
**24**	L-in18-2-1	CAATCAAATTCCACTCATTCC
**25**	L-in18-1-1	TACATTCTCACTTAAATACTCTACTC
**26**	L- in18-0	CATCTCAATCCTCATCAACTC

Supplementary Table 5. Parameters used in kinetic simulations

Parameter	value	Description
**K(s)**	0.01	Reaction rate with switching signal
**K(c)**	0.01	Reaction rate with current signal
**K(l1)**	1.0E-4	Leakage rate of blocked switch
**K(l2)**	5.0E-5	Leakage rate of OFF-state switch
**switch**	1	Initial concentration of switches (gates)
**input**	1	Concentration of switching signal (input for logic gate)

Supplementary References

1. Qian, L. & Winfree, E. Scaling Up Digital Circuit Computation with DNA Strand Displacement Cascades. *Science* **332**, 1196-1201 (2011).
2. Seelig, G., Soloveichik, D., Zhang, D.Y. & Winfree, E. Enzyme-free nucleic acid logic circuits. *Science* **314**, 1585-1588 (2006).
3. Li, W., Zhang, F., Yan, H. & Liu, Y. DNA based arithmetic function: a half adder based on DNA strand displacement. *Nanoscale* **8**, 3775-3784 (2016).
